# A Randomized Trial of Precision Prevention Materials to Improve Primary and Secondary Melanoma Prevention Activities among Individuals with Limited Melanoma Risk Phenotypes

**DOI:** 10.3390/cancers13133143

**Published:** 2021-06-23

**Authors:** John Charles A. Lacson, Scarlet H. Doyle, Lu Qian, Jocelyn Del Rio, Stephanie M. Forgas, Stella Valavanis, Rodrigo Carvajal, Guillermo Gonzalez-Calderon, Youngchul Kim, Richard G. Roetzheim, Steven K. Sutton, Susan T. Vadaparampil, Peter A. Kanetsky

**Affiliations:** 1 Department of Cancer Epidemiology, H. Lee Moffitt Cancer Center & Research Institute, Tampa, FL 33612, USA; JohnCharles.Lacson@moffitt.org (J.C.A.L.); scarletdoyle93@gmail.com (S.H.D.); Jocelyn.DelRio@moffitt.org (J.D.R.); Stephanie.Forgas@moffitt.org (S.M.F.); Stella.Valavanis@moffitt.org (S.V.); 2 SWOG Statistics & Data Management Center, Fred Hutchinson Cancer Research Center, Seattle, WA 98109, USA; lqian@fredhutch.org; 3 Biostatistics and Bioinformatics Shared Resources, H. Lee Moffitt Cancer Center and Research Institute, Tampa, FL 33612, USA; rodrigo.carvajal@moffitt.org (R.C.); Guillermo.Gonzalez-Calderon@moffitt.org (G.G.-C.); 4 Department of Biostatistics and Bioinformatics, H. Lee Moffitt Cancer Center and Research Institute, Tampa, FL 33612, USA; Youngchul.Kim@moffitt.org (Y.K.); Steve.Sutton@moffitt.org (S.K.S.); 5 Department of Family Medicine, Morsani College of Medicine, University of South Florida, Tampa, FL 33612, USA; roetzheim@usf.edu; 6 Department of Outcomes and Behavior, H. Lee Moffitt Cancer Center and Research Institute, Tampa, FL 33612, USA; Susan.Vadaparampil@moffitt.org

**Keywords:** randomized trial, intervention trial, melanoma, prevention, sun-resistant, *MC1R*, precision prevention, genetic testing, public health genetics

## Abstract

**Simple Summary:**

Inherited genetic variation at the *MC1R* gene is associated with increased risk of melanoma among non-Hispanic whites (NHWs), especially among those with skin and pigmentation characteristics that are associated with average to lower melanoma risk, for whom *MC1R* genetic testing may reveal unrecognized melanoma risk. We conducted a randomized trial to examine whether providing *MC1R* genetic risk information together with precision prevention materials would increase primary and secondary melanoma preventive behaviors compared to providing generic prevention materials only. We found that among participants with *MC1R* variants associated with higher risk of melanoma, the intervention increased shade-seeking or using an umbrella, increased wearing sleeved shirts, and decreased sunburns among their young children. We conclude that *MC1R* genetic testing and precision prevention materials may increase the practice of some sun-protective behaviors.

**Abstract:**

Inherited variation at *MC1R* is associated with elevated melanoma risk among non-Hispanic whites (NHWs). *MC1R* genetic testing may unmask previously unrecognized disease risk, especially among individuals with few melanoma phenotypic risk factors. We recruited NHW individuals with limited phenotypic risk factors from two primary care clinics in west-central Florida. Participants (*n* = 1134) were randomized within *MC1R* genotype risk group (average/higher) to receive mailed precision prevention (i.e., intervention) or generic prevention materials. Participants reported hours of weekday and weekend sun exposure, frequency of intentional outdoor tanning and sun protection behaviors, number of sunburns, indoor tanning episodes, and skin examinations at baseline, and after 6 and 12 months. Among *MC1R* higher-risk participants, the intervention increased the likelihood of often or always wearing a shirt with sleeves (OR = 1.49, *p* = 0.03) and seeking shade or using an umbrella (OR = 1.42, *p* = 0.046), and it decreased the number of sunburns among their young children (β = −0.13, *p* = 0.03). Intervention effects were not noted among *MC1R* average-risk participants. Moderation analyses identified intervention effects within subgroups in average-risk and higher-risk participants. Precision prevention information conveying *MC1R* testing results can increase the practice of some sun protection behaviors among at-risk individuals with limited melanoma risk phenotypes and may provide a cross-generational tool to counteract increasing incidence of melanoma.

## 1. Introduction

Incidence of cutaneous malignant melanoma has increased over the past 50 years in populations with predominantly European ancestry [1,2]. Exposure to ultraviolet radiation, especially intermittent sun exposure resulting in sunburn, is the main environmental factor associated with increased melanoma risk [3]. Primary prevention strategies to reduce melanoma risk include wearing sunscreen and sun-protective clothing, and minimizing sun exposure and intentional tanning [4,5,6]. Secondary prevention activities to detect melanoma at an early stage when surgically curable include skin examinations performed by oneself, partner, or health provider [6,7,8]. These prevention activities are not well-practiced across the population [9,10].

Inherited genetic variation at the melanocortin-1 receptor (*MC1R*) gene is a robust risk marker for melanoma among individuals of European ancestry [11,12]. The nine most prevalent *MC1R* variants range in frequency from about 0.5 to 11% among these individuals, with each variant imparting a 1.5- to 2.7-fold increased odds of melanoma [12]. The risk conferred by *MC1R* variants is notably stronger among individuals with phenotypes associated with average to lower melanoma risk, including those with darker natural hair color, those who tan well, do not severely burn, and/or develop fewer freckles after sun exposure [12,13]. Attributable risk is a construct that quantifies the theoretical disease reduction possible through the “removal” of an exposure. Among individuals with sun-resistant phenotypic traits, the amount of melanoma risk attributed to the carriage of one of the nine most prevalent *MC1R* variants, ranging from 0.4 to 15%, and the attributable risk summed across the nine variants is estimated between 37 and 45% [12,14]. Thus, for individuals with phenotypes associated with lower to average melanoma risk, *MC1R* genetic testing can reveal information about melanoma risk not otherwise deduced from clinical observation alone, has the potential to unmask at-risk subgroups in this population who may be unaware of the constitutional risk imparted by their genetic inheritance, and can impact overall melanoma rates [13].

In this randomized controlled trial, we examined whether receipt of precision prevention information communicating results of *MC1R* genotyping can improve sun-related behaviors among individuals with phenotypes that are associated with lower to average melanoma risk. We assessed intervention effects on sun-related behaviors and skin examination and tested for moderation by baseline characteristics. Our precision prevention materials were anchored in Protection Motivation Theory, which posits that the higher the perceived vulnerability of developing disease, the more likely an individual is to adopt preventive behaviors, as long as these behaviors are effective in eliminating the threat and the individual believes themselves to be capable of adopting such behaviors [15]. Thus, we hypothesized that an intervention effect would be observed among those at higher genetic risk for melanoma, but not among those at average risk. In exploratory analyses, we assessed the impact of the precision prevention intervention on sun-related behaviors among young children of study participants.

## 2. Materials and Methods

### 2.1. Setting and Participants

Participants were recruited from two university-associated primary care clinics in Florida between September 2015 and September 2018. Participant data were captured and managed throughout the study using an in-house HIPAA-compliant web-based database management system. Inclusion criteria were: (1) non-Hispanic, (2) white, (3) at least 18 years of age, and (4) fluent in English. Exclusion criteria included: (1) report of a full-body skin examination within the past year and (2) personal history of melanoma.

Participants were required to have brown/black natural hair color at 18 years old and at least three of the following criteria: (1) mild to no freckling at summer’s end; (2) brown or black natural eye color; (3) mild to no burning after exposure to a first strong summer sun; and (4) medium to dark tan after prolonged sun exposure. This phenotypic eligibility structure assured that participants had only limited phenotypic risk characteristics; individuals with Fitzpatrick skin type I [16], the most sun-sensitive skin type, were systematically considered ineligible. This structure did result in the inclusion of individuals who reported one risk phenotype, e.g., painful or severe burning after exposure to the sun for the first time during summer, as long as it was reported in the absence of other phenotypic risk factors. Starting in December 2016, eye color was removed from the eligibility criteria to increase the number of potentially eligible participants. Patients with subsequent clinic appointments previously ineligible because of eye color and had at least two of the three remaining criteria were re-approached.

This protocol was registered in April 2018 on clinicaltrials.gov (NCT03509467).

### 2.2. Biospecimens and Genotyping

Saliva samples were collected using Oragene kits (DNA Genotek, Inc., Ottawa, Canada). Standard procedures were followed to extract germline DNA, PCR-amplify, and sequence the 951 bp one-exon region of the melanocortin-1 receptor (*MC1R*) gene. A list of observed *MC1R* variants and corresponding risk level is presented in Supplementary Table S1. Variants were classified as higher-risk based on elevated odds (OR ≥ 1.80) of melanoma among individuals with limited risk phenotypes [12] or having an HVAR score > 0.909 as determined by the bioinformatic tool Polyphen [17]. Participants were categorized as higher-risk if they carried at least one higher-risk variant, otherwise, they were average-risk.

### 2.3. Randomization and Mailed Prevention Materials

Participants who completed the baseline questionnaire and for whom *MC1R* genotyping was successful (*n* = 1134) were block-randomized within *MC1R* risk group into the precision prevention or standard arm. Precision prevention materials were adapted from Hay and colleagues [18,19] and were developed to minimize health literacy and health numeracy demands. Participants in the precision prevention arm received mailed materials containing information on: (1) melanoma and skin cancer; (2) genetic risk for melanoma; (3) *MC1R* and its role in developing melanoma, their *MC1R* risk group, and interpretation of risk information; and (4) melanoma prevention behaviors in the context of genetic risk. Participants in the standard arm received mailed materials containing information on (1) melanoma and skin cancer; and (2) melanoma prevention behaviors recommended by the American Academy of Dermatology. All participants received information on sun protection behaviors targeted at children.

**Figure 1:**
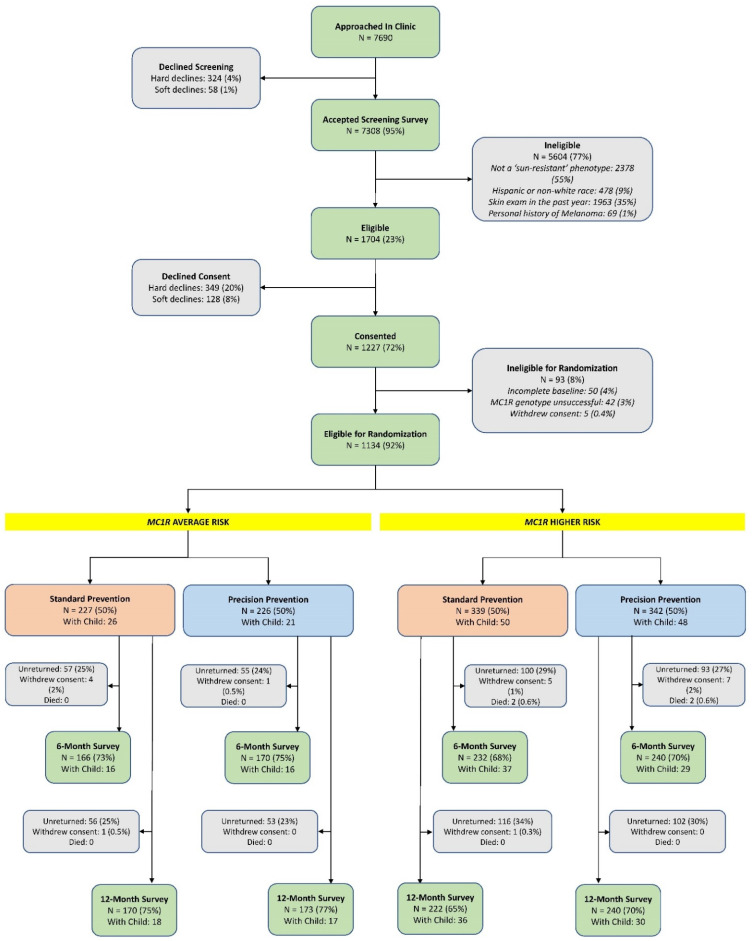
Flow diagram of the parallel randomized intervention trial.

Participants for whom genotyping failed (*n* = 42) were excluded from the study (Figure 1). These individuals were sent a letter stating their sample was unable to be successfully processed. Enclosed with the letter was a USD 10 gift card in gratitude for their participation and generic skin cancer prevention materials targeted at adults and children.

Within two weeks of mailing prevention materials, telephone follow-ups were initiated to confirm receipt of intervention materials and answer participant’s questions, but not to proactively reiterate or reinforce any aspect of the prevention materials. After follow-up or three unsuccessful attempts, participants were sent a summary letter that briefly summarized prevention materials (all participants) and their *MC1R* risk group (intervention arm). Participants were incentivized with a USD 20 gift card after telephone follow-up, and another USD 20 gift card upon completing the 12-month survey.

### 2.4. Study Assessments

A baseline assessment elicited information on age, gender, marital status, education, health literacy, health numeracy, and family history of melanoma and non-melanoma skin cancer (Table 1). Health literacy was measured by asking “How confident are you filling out medical forms by yourself?” (not at all, a little bit, somewhat, quite a bit, extremely) [20]. Health numeracy was determined by asking “In general, how easy or hard do you find it to understand medical statistics?” (very easy, easy, hard, very hard) [21]. The baseline questionnaire also assessed work outdoors, family history of other (non-skin) cancers, and a variety of psychosocial constructs (Supplementary Table S2).

Participants reported on seven prevention outcome activities over the past 12 months: (1) number of hours spent outside between 10 a.m. and 4 p.m. separately for weekdays and weekends; (2) number of severe or painful sunburns; (3) frequency (never, rarely, sometimes, often, always) of each of five sun protection behaviors: wearing a shirt with sleeves, a hat, and sunglasses, seeking shade or using an umbrella while outside, and using sunscreen; (4) frequency (never, rarely, sometimes, often, always) of intentional outdoor tanning; (5) number of intentional indoor tanning occurrences; (6) skin examination (yes/no) performed by a health provider; and (7) number of skin examinations performed by oneself or partner. These questions were taken from a standardized survey of sun exposure and sun protection behaviors [22]. In addition, melanoma cancer worry was assessed using a 3-item adaptation of the Lerman cancer worry scale [23,24].

Participants with at least one child aged 10 years or younger completed additional questions eliciting information on the child’s age, gender, untanned skin color, and on primary prevention activities of the child, except indoor tanning.

Participants completed a 6- and 12-month survey that reassessed all outcome measures over the past 6 months. If a participant did not return their 6-month questionnaire, their 12-month assessment specified a 12-month period.

### 2.5. Statistical Analysis

All analyses were conducted in parallel within the average- and higher-risk categories using SAS (ver. 9.4, SAS Institute, Cary, NC, USA). For each outcome, we used generalized estimating equations (GEEs) to assess the impact of the precision prevention intervention by simultaneously modeling the 6- and 12-month outcome measures and adjusting for covariates. Because participants were randomized continuously throughout the year and melanoma prevention behaviors are seasonal, the predicted population marginal means of 6- and 12-months outcome measures were averaged to represent one post-intervention assessment. Statistically significant intervention effects were defined as having a type III *p*-value ≤ 0.05, which tests the departure from the null hypothesis across all four cross-product terms of time and study arm. Because type III *p*-values are distinct from those testing the departure from the null of a point estimate, 95% confidence intervals around individual beta’s are not provided.

Randomization was assessed by univariate comparisons (i.e., two-sample *t*-test, Wilcoxon rank-sum test, chi-squared homogeneity test) of baseline variables between the intervention and standard arms. Any variable with *p* ≤ 0.05 was included as a covariate in GEE models. To preserve GEE’s robustness to missingness, which assumes missingness at random, we conducted univariate logistic regression analyses for all baseline measures (Table 1 and Supplementary Table S2) to identify predictors of missingness (*p* ≤ 0.10) separately for the 6- and 12-month timepoints, followed by a backward stepwise selection on these variables to obtain a parsimonious set of baseline missingness predictors (*p* ≤ 0.05) to include as covariates (Supplementary Table S3). We used a similar strategy to identify significant baseline predictors of each outcome, which were included as covariates along with the baseline outcome and season at baseline (spring, summer, fall, or winter).

Weekday and weekend sun exposure, number of sunburns, and frequency of outdoor intentional tanning were assumed to have a normal distribution and were modeled using the canonical identity link function. The five component sun protection behaviors were also examined individually as a repeated binary outcome (often or always vs. sometimes, rarely, or never) and modeled using a logit link function. Due to low indoor tanning prevalence in our sample, multivariate models of indoor tanning were unstable, precluding estimation of an intervention effect—raw proportions are reported.

We transformed measures of skin examination into dichotomous variables to reflect ever having a skin examination over the study period. Separate models were used to assess skin examinations conducted by a health professional, by oneself or a partner, and by either a health professional or oneself or a partner. We used logistic regression models to determine odds ratios (ORs) and 95% confidence intervals (CIs) for skin exams. Although report of a full-body skin exam within the past year was an exclusion criterion, 25 participants reported having a skin exam between screening and completion of the baseline questionnaire and were excluded from the analysis of professional skin exams. Similarly, those who had either a professional or self/partner skin exam (*n* = 135) between screening and baseline were excluded from the analysis of self/partner skin exams. Analyses of skin exams were restricted to participants who returned the 12-month survey. Predictors of missingness were not included as covariates in these analytic models.

To estimate changes in each outcome between baseline and post-intervention within each arm, we constructed distinct GEE models that included the baseline outcome measure as a dependent variable rather than as a covariate.

For each outcome, we tested for moderation of the intervention effect by assessing the statistical significance of an interaction term between the moderator and the study arm. A priori moderators included age (continuous), education (5-level ordinal), marital status (married, civil union, domestic partnership vs. single, divorced, separated, widowed), gender (female/male), and family history of melanoma and non-melanoma skin cancer (yes/no). We also tested tendency to burn as a moderator by categorizing individuals who had moderate to severe sunburns after acute sun exposure as burners and those who had no or only mild sunburns as non-burners. Because less than 10% of our study sample reported lower health literacy (not at all, a little bit, somewhat (confident in filling out medical forms independently)) or lower health numeracy (hard or very hard (to understand medical statistics)), it was not possible to assess these variables as moderators.

## 3. Results

A total of 1704 (23%) screened individuals were eligible for the study; 1227 (72%) provided informed consent (Figure 1). After removing 93 individuals with incomplete baseline questionnaires, unsuccessful genotyping, or who withdrew consent, 1134 individuals were randomized. There were 32 individuals whose baseline surveys were received at least one week after mailing their intervention materials. These participants were included in our primary intent-to-treat analyses reported herein but were removed in secondary per protocol analyses. Among randomized participants, 808 (71.3%) and 805 (71.0%) completed the 6- and 12-month surveys, respectively (Figure 1). There were minimal differences in baseline characteristics by study arm (Table 1).

### 3.1. MC1R Average-Risk Participants

Comparing post-intervention to baseline measures, we noted statistically significant reductions in weekday sun exposure, sunburns, and outdoor intentional tanning among standard arm participants (*p* < 0.05, Table 2). Among participants on the intervention arm, we observed statistically significant reductions in weekend sun exposure, sunburns, and outdoor intentional tanning and increases in wearing a hat and seeking shade or using an umbrella (*p* < 0.05). Melanoma cancer worry statistically significantly decreased in both the intervention (*p* < 0.0001) and standard arms (*p* < 0.0001).

The intervention was not associated with a change in any primary prevention outcome (Table 2), skin examination measurement (Table 3), or melanoma cancer worry (β = 0.004, *p* = 0.94).

Family history of melanoma was a moderator of the intervention effect on wearing a hat often or always (*p* = 0.03) and seeking shade or using an umbrella often or always (*p* = 0.02, Table 4). Those in the intervention arm with a family history were statistically significantly more likely to wear a hat (OR = 5.23, *p* = 0.01) and seek shade or use an umbrella often or always (OR = 3.67, *p* = 0.01), while those without a family history had no change (hat OR = 1.05, *p* = 0.87; shade/umbrella OR = 0.98, *p* = 0.93), compared to those in the standard arm. Family history of non-melanoma skin cancer was a moderator of the intervention effect on number of sunburns (*p* = 0.049, Table 4). Those with a family history exhibited non-significant increases in sunburns (β = 0.09, *p* = 0.23), while those without a family history tended to report decreases in sunburns (β = −0.10, *p* = 0.07). Marital status was a statistically significant moderator of the intervention effect on weekend sun exposure (*p* = 0.019, Table 4). Single, separated, divorced, or widowed participants decreased their weekend sun exposure (β = −0.15, *p* = 0.34), while participants in a marriage, domestic partnership or civil union increased their weekend sun exposure (β = 0.32, *p* = 0.01).

### 3.2. MC1R Higher-Risk Participants

We noted statistically significant improvements (*p* < 0.05) in weekday and weekend sun exposure, sunburns, outdoor intentional tanning and wearing a hat when comparing post-intervention to baseline measures within both intervention and standard arms, and in seeking shade or using an umbrella within the intervention arm (Table 2). Melanoma cancer worry statistically significantly decreased in both the intervention (*p* < 0.0001) and standard arms (*p* < 0.0001).

When comparing outcomes among participants on the intervention and standard arms, the intervention statistically significantly increased the likelihood of often or always seeking shade or using an umbrella (OR = 1.42, *p* = 0.046) and wearing sleeved shirts (OR = 1.49, *p* = 0.033, Table 2). There were no intervention effects on skin examinations (Table 3). In addition, the intervention statistically significantly decreased melanoma cancer worry (β = −0.05, *p*=0.01).

Tendency to burn was a statistically significant moderator of the intervention effect on the number of sunburns among *MC1R* higher-risk participants (*p* = 0.032, Table 4). Burners decreased their sunburns (β = −0.14, *p* = 0.09), while non-burners had a slight increase in sunburns (β = 0.07, *p* = 0.16).

### 3.3. Children of Participants

Forty-six participants in the *MC1R* average-risk group had at least one child 10 years old or younger at baseline (mean child age = 3.11, SD = 0.91) as did 95 participants in the *MC1R* higher-risk group (mean child age = 3.18, SD = 0.82) (Figure 1).

Among *MC1R* average-risk participants, we found no differences in any outcomes reported for children between post-intervention and baseline, regardless of intervention arm (Table 4), nor did we find any intervention effects on any outcomes. Among *MC1R* higher-risk participants, there was a statistically significant reduction in outdoor tanning among children between baseline and post-intervention measures (*p* = 0.02, Table 5) for those in the standard arm. In the intervention arm, there was a decrease in average number of sunburns (*p* = 0.01) reported for children. There was a statistically significant intervention effect on child sunburns (β = −0.13, *p* = 0.03).

### 3.4. Per Protocol Analyses

The results from our per protocol analyses are consistent with those from the intent-to-treat analyses.

## 4. Discussion

Among participants who inherited *MC1R* higher-risk variants, we found our precision prevention intervention increased their likelihood to wear a hat and seek shade or use an umbrella often or always. However, the intervention did not impact secondary prevention outcomes in this group. We also noted a decrease in number of sunburns among younger children of *MC1R* higher-risk participants attributed to the precision prevention intervention. In contrast, among *MC1R* average-risk participants, we did not observe any main intervention effects on sun-related outcomes or skin examinations, or among outcomes in their children. These findings support our hypothesis that feedback of precision prevention information would be salient only among participants who received materials conveying their increased inherited genetic risk of melanoma. We did not observe increases in melanoma risk behaviors among average-risk participants, dispelling worries that communicating low or average risk may lead to a false sense of security and an increase in risky behavior [25].

Our finding of an increased likelihood to practice sun-protective behaviors among participants receiving precision prevention materials is consistent with findings from other studies showing improvements in selected primary and secondary melanoma prevention behaviors [26,27,28]. However, these studies enrolled individuals from families with hereditary melanoma, examined the receipt of genetic testing results in the highly penetrant *CDKN2A* gene, and provided genetic counseling in the intervention arms. Our study shows that feedback of genetic information on a low–moderate penetrance gene, *MC1R*, in the format of a mailed packet and without formal genetic counseling, can lead to an improvement in some melanoma prevention behaviors, and highlights the plausibility of a public health genomics approach to influence skin cancer risk-reducing behaviors at the population-level. This finding is congruent with a systematic review and meta-analysis of 17 studies on the behavioral impact of genetic testing for complex diseases, which reported statistically significant increases in self-reported behavior change 6 months or later after results return among risk variant carriers compared to non-carriers [29].

Specifically, we noted statistically significant intervention effects on both seeking shade or using an umbrella and wearing sleeved shirts. It is possible that heightened use of shade may counterbalance movement toward decreasing the number of hours spent outdoors, thus reducing our ability to detect an intervention effect on sun exposure. Both increased use of shading while outside and wearing a sleeved shirt also could lead to a reduction in the number of sunburns; and we observed an intervention effect on decreased sunburns among burners, i.e., the subgroup of participants who reported their unprotected skin would develop a painful or severe sunburn after exposure to one hour of sunlight for the first time in summer, yet who also reported having naturally dark hair, a proclivity to moderately or deeply tan, and few or no freckles. However, this intervention effect was not observed among non-burners possibly because these individuals had fewer sunburns and thus less ability to improve this outcome. At baseline, burners averaged 0.83 severe or painful sunburns (SD = 1.10) within the past year, while non-burners averaged 0.65 sunburns (SD = 0.96).

Surprisingly, we observed intervention effects among selected subgroups of *MC1R* average-risk participants. Among those reporting a family history of melanoma, the precision prevention intervention improved hat-wearing and shade-seeking or umbrella use, findings similar to those observed among *MC1R* higher-risk participants. Perhaps among this subgroup of individuals, messaging regarding genetic risk, even if not elevated, reinforces the tie between inherited genetics and family history to motivate positive behavior change. In contrast, the intervention increased the number of sunburn episodes and increased average weekend hours in the sun among participants with a family history of non-melanoma skin cancer and among participants with a legally-recognized partner, respectively. These unexpected findings warrant further investigation to better understand factors mediating these intervention effects, in particular to guard against the incorrect perception that receipt of *MC1R* average-risk information translates to no risk of developing melanoma.

We found no intervention effect on skin examinations, which may partially be due to the study setting and recruiting patients with upcoming primary care appointments. Screening and consenting participants before or during their clinic appointments may have encouraged them to seek a skin examination from their provider or discuss the benefits of regular self or partner skin exams, indicative of the Hawthorne effect [30]. Our clinic-based setting also may partially explain why some participants reported absence of a professional skin examination upon screening but indicated on the baseline assessment having undergone a skin exam.

We noted post-intervention improvements in most prevention activities across all study participants, regardless of intervention arm or *MC1R* risk group. Thus, providing even generic information on melanoma risk-reducing behaviors resulted in multiple positive behavior changes. A future study that directly tests the receipt of precision or generic prevention materials compared to the status quo, i.e., receipt of no prevention information, would provide more definitive information on the comparative utility of these intervention approaches.

We also found that melanoma worry decreased among participants regardless of study arm or *MC1R* risk category; and that among *MC1R* high-risk intervention participants, those receiving the precision prevention materials had statistically significantly lower post-intervention melanoma worry than those in the standard arm. These findings suggest that while receipt of any type of melanoma prevention information may serve to alleviate melanoma worry in participants, receipt of precision information may better help to dispel melanoma worry.

Despite small numbers and not explicitly informing intervention arm participants that their genetic risk may be shared by other blood relatives, we observed an intervention effect on the number of sunburns among children of *MC1R* higher-risk participants. Previous studies have shown that parents who had sunburns or a positive attitude toward tanned skin tend to have children with increased occurrences of sunburns, intentional indoor and outdoor tanning, and other sun-seeking behaviors [31,32,33,34]. Although contrasting in influencing melanoma promotion or risk behaviors, these findings suggest that a “cross-generational” approach may be a mechanism to reduce the burden of melanoma through parent’s prompting of primary prevention activities in their children. Future research should examine sun-exposure activities in teenage children, because both childhood and adolescence are critical windows of exposure for melanoma risk [35,36], and studies have shown suboptimal sun-protective behaviors among adolescents [37,38], even among those with a family history of melanoma [39,40,41,42].

One limitation of our study was modest completion rates (65–77%) of our 6- and 12-month assessments, which reduced statistical power to detect small differences. Additionally, because study enrollment occurred throughout the year and many sun-related activities are seasonal, even in Florida, it was inappropriate to separately analyze 6- and 12-month intervention effects. A participant’s 6-month outcomes may depend on the time they start the study, e.g., changes reported from April to September may be different to changes reported from October to March. An alternative study design is to limit study enrollment to one season, circumventing this issue by aligning the timing of the intervention and assessments across all participants. We also were unable to examine changes in sun-protective behavior beyond 12 months. To achieve meaningful reductions in risk, individuals must retain long-term habits of sun protection, UVR avoidance, and undergo routine skin exams. Although our intervention materials were developed to minimize health literacy and health numeracy demands, because of the high levels of education, health literacy, and health numeracy in our study population, our results may have limited generalizability to populations with lower education and socioeconomic levels. We also acknowledge that measurement of phenotypic traits informing study eligibility and outcome measurement was based on self-reports. Finally, after controlling the false discovery rate [43], none of our findings retained statistical significance. However, because melanoma prevention activities are correlated, correction for multiple hypotheses testing results in overly conservative *p*-value thresholds.

Despite our findings of a main intervention effect on two sun-protective behaviors, our target population may have prevented us from observing intervention effects on other primary prevention outcomes. Individuals with phenotypes associated with lower to average melanoma risk may have less agency to change their sun-related behavior because of the lack of immediate negative physical responses (sunburn, reddening, etc.) that would otherwise indicate excessive sun exposure and/or inadequate sun protection. However, since the risk imparted by *MC1R* variants is stronger in or exclusive to these individuals [12,13], we aimed to target the population who may benefit the most from *MC1R* genetic testing by providing risk information that would otherwise be unavailable.

## 5. Conclusions

This intervention trial demonstrates that receipt of melanoma precision prevention material anchored in *MC1R* testing results can improve selected sun-protective behaviors among adults with phenotypes that are associated with lower to average melanoma risk who inherit higher-risk *MC1R* variants, as well as among subgroups of individuals reporting a tendency to burn and reporting a family history of melanoma who inherit higher-risk *MC1R* variants. Our finding of a decreased number of sunburns among children of *MC1R* higher-risk participants who received precision prevention materials warrants further research to examine effects on children and adolescents. Additional research is also needed to explore genetic risk knowledge retention and long-term adherence to improved sun-protective behaviors, and the salience of a precision prevention approach among individuals with lower educational attainment, health literacy, and health numeracy.

## Figures and Tables

**Table 1:** Baseline characteristics of the study population.

	*MC1R* Average-Risk, *n* (%)			*MC1R* Higher-Risk, *n* (%)		
Variable	Standard Arm (*n* = 227)	Precision Prevention Arm (*n* = 226)	*p*-Value *	Standard Arm (*n* = 339)	Precision Prevention Arm (*n* = 342)	*p*-Value *
***Participant Characteristics***						
**Age (mean, SD)**	48.1 (16.0)	46.6 (16.8)	0.34	48.5 (15.9)	47.7 (14.9)	0.55
**Female**	112 (49.3)	116 (51.3)	0.67	175 (51.6)	168 (49.1)	0.51
**Marital status**			0.79			0.06
Single or never married	59 (26.0)	60 (26.5)		67 (19.8)	79 (23.1)	
Married, domestic partnership, or civil union	138 (60.8)	134 (59.3)		228 (67.3)	201 (58.8)	
Divorced, separated, or widowed	27 (11.9)	32 (14.2)		42 (12.4)	59 (17.3)	
**Education**			0.80			0.13
Graduate degree or higher	70 (30.8)	66 (29.2)		102 (30.1)	108 (31.6)	
Four-year college degree	55 (24.2)	61 (27.0)		109 (32.2)	122 (35.7)	
Some college †	47 (20.6)	49 (21.6)		62 (18.3)	53 (15.5)	
High school or GED	45 (19.8)	41 (18.1)		53 (15.6)	45 (13.2)	
Less than high school or GED	7 (3.1)	9 (4.0)		11 (3.2)	10 (2.9)	
**Season at baseline**			0.92			0.84
Spring	71 (31.3)	66 (29.2)		89 (26.3)	93 (27.2)	
Summer	39 (17.2)	38 (16.8)		60 (17.7)	63 (18.4)	
Fall	61 (26.9)	60 (26.5)		106 (31.3)	96 (28.1)	
Winter	56 (24.7)	62 (27.4)		84 (24.8)	90 (26.3)	
**Health literacy**			0.94			0.91
Extremely confident	151 (66.5)	153 (67.7)		233 (68.7)	230 (67.3)	
Quite a bit confident	57 (25.1)	47 (20.8)		75 (22.1)	85 (24.9)	
Not at all, a little bit, or somewhat confident	17 (7.5)	25 (11.1)		31 (9.1)	25 (7.3)	
**Health numeracy**			0.004			0.26
Very easy	121 (53.3)	92 (40.7)		157 (46.3)	142 (41.5)	
Easy	91 (40.1)	110 (48.7)		163 (48.1)	178 (52.0)	
Hard	12 (5.3)	22 (9.7)		18 (5.3)	18 (5.3)	
Very hard	1 (0.4)	1 (0.4)		0	1 (0.3)	
**Family history of melanoma**	38 (16.7)	47 (20.8)	0.28	74 (21.8)	74 (21.6)	0.94
**Family history of skin cancer**	68 (30.0)	61 (27.0)	0.52	97 (28.6)	99 (28.9)	0.92
**Burnability**			0.75			0.63
Burner	49 (21.6)	46 (20.4)		112 (33.0)	119 (34.8)	
Non-burner	178 (78.4)	180 (79.7)		227 (67.0)	223 (65.2)	
***Outcomes***						
**Sun exposure (hours) (mean, SD)**						
Weekday	1.4 (1.3)	1.4 (1.0)	0.97	1.3 (1.1)	1.4 (1.3)	0.15
Weekend	2.3 (1.5)	2.4 (1.4)	0.58	2.1 (1.4)	2.4 (1.6)	0.05
**Sunburns (mean, SD)**	0.6 (1.0)	0.7 (1.0)	0.43	0.7 (1.0)	0.8 (1.1)	0.23
**Outdoor intentional tanning (mean, SD)**	1.9 (1.0)	2.0 (0.9)	0.48	2.0 (1.0)	2.0 (1.0)	0.58
**Indoor tanning**	10 (0.04)	12 (0.05)	0.63	18 (0.05)	7 (0.02)	0.03
**Wearing a hat often or always**	62 (27.6)	56 (25.2)	0.58	92 (27.2)	95 (28.0)	0.81
**Seeking shade or using umbrella often or always**	86 (38.2)	71 (31.8)	0.16	103 (30.5)	106 (31.2)	0.84
**Wearing a shirt with sleeves often or always**	158 (69.9)	154 (69.1)	0.84	220 (65.1)	228 (66.9)	0.63
**Wearing sunglasses often or always**	161 (71.6)	153 (68.6)	0.50	217 (64.4)	233 (68.5)	0.25
**Wearing sunscreen often or always**	82 (36.3)	66 (29.6)	0.13	111 (32.8)	127 (37.2)	0.23

* *p*-values are from t-tests for normally distributed variables, Wilcoxon rank-sum tests for ordinal and non-normally distributed variables, and chi-squared tests for categorical variables. † Participants who indicated they received their education outside of the U.S. were assigned to the median value (some college).

**Table 2:** Primary prevention outcome measures at baseline and post-intervention and intervention effects by *MC1R* risk category.

	Standard Arm			Precision Prevention Arm			Intervention Effect *	
Outcomes	Baseline	Post-Intervention ^†^	*p* ^‡^	Baseline	Post-Intervention ^†^	*p* ^‡^	Beta/Odds Ratio	*p*
*MC1R* Average-Risk								
	*n* = 227			*n* = 226				
**Continuous Outcomes ^§^**								
Weekday hours	1.27	1.09	0.009	1.30	1.18	0.069	0.12	0.16
Weekend hours	2.17	2.00	0.067	2.25	2.08	0.031	0.11	0.18
Sunburns	0.60	0.29	<0.0001	0.66	0.20	<0.0001	-0.07	0.35
Outdoor intentional tanning	2.01	1.79	<0.0001	2.03	1.85	<0.0001	0.06	0.48
**Binary Outcomes ^¶^**								
Wearing a hat often or always	0.20	0.24	0.09	0.17	0.29	<0.0001	1.36	0.25
Seeking shade or using umbrella often or always	0.35	0.34	0.89	0.27	0.35	0.02	1.24	0.32
Wearing a shirt with sleeves often or always	0.72	0.71	0.79	0.70	0.72	0.59	1.08	0.72
Wearing sunglasses often or always	0.76	0.77	0.61	0.75	0.76	0.57	0.91	0.72
Wearing sunscreen often or always	0.36	0.37	0.98	0.28	0.30	0.53	0.85	0.51
Indoor intentional tanning	0.05	0.01	--	0.06	0.03	--	--	--
*MC1R* Higher-Risk								
	*n* = 339			*n* = 342				
**Continuous Outcomes ^§^**								
Weekday hours	1.26	1.09	0.001	1.29	1.05	<0.0001	0.01	0.74
Weekend hours	2.25	1.89	0.002	2.30	1.89	0.001	-0.01	0.76
Sunburns	0.83	0.27	<0.0001	0.85	0.28	<0.0001	0.03	0.97
Outdoor intentional tanning	1.96	1.70	<0.0001	1.95	1.71	<0.0001	0.004	0.98
**Binary Outcomes ^¶^**								
Wearing a hat often or always	0.25	0.33	0.001	0.27	0.32	0.03	0.87	0.53
Seeking shade or using umbrella often or always	0.33	0.35	0.48	0.34	0.43	0.006	1.42	0.046
Wearing a shirt with sleeves often or always	0.68	0.67	0.83	0.70	0.75	0.08	1.49	0.033
Wearing sunglasses often or always	0.66	0.70	0.16	0.70	0.73	0.23	1.13	0.61
Wearing sunscreen often or always	0.36	0.42	0.10	0.40	0.38	0.44	0.83	0.39
Indoor intentional tanning	0.06	0.03	--	0.02	0.01	--	--	--

* Intervention effect compares the post-intervention measure in the intervention arm to that in the standard arm, after adjusting for baseline outcome, season, predictors of missingness, and predictors of the outcome. ^†^ Post-Intervention is the average of outcome measures obtained at the 6- and 12-month assessments. ^‡^ Within arm *p*-values are from tests comparing post-intervention measures to baseline averages from a GEE model containing baseline, 6-, and 12-month outcomes as the dependent variables. **^§^** Baseline and post-intervention values are population predicted marginal means, while the intervention effects are beta-coefficients. ^¶^ Baseline and post-intervention values are population predicted marginal proportions, while the intervention effects are reported as odds ratios (ORs). For indoor intentional tanning, only raw (unadjusted) proportions of participants who underwent indoor tanning are reported.

**Table 3:** Secondary prevention outcomes at post-intervention and intervention effects by *MC1R* risk category.

Skin Exam Type	Standard Arm		Precision Prevention Arm		Intervention Effect *			
	*n* ^†^	Post- Intervention ^‡^	*n* ^†^	Post- Intervention ^‡^	Odds Ratio	95% Confidence Interval		*p*
*MC1R* Average-Risk								
Health professional	166	0.18	169	0.17	0.90	0.51	1.60	0.72
Self/partner	153	0.17	148	0.19	1.17	0.64	2.11	0.61
Either	151	0.33	144	0.34	1.02	0.62	1.69	0.94
*MC1R* Higher-Risk								
Health professional	216	0.21	229	0.21	0.96	0.60	1.52	0.86
Self/partner	202	0.28	211	0.23	0.76	0.49	1.20	0.24
Either	199	0.37	202	0.38	1.01	0.66	1.53	0.98

* Intervention effect compares the post-intervention measure in the intervention arm to that in the standard arm, after adjusting for season and baseline predictors of outcome. ^†^ Participants who reported having had a skin examination completed by a health professional at the baseline assessment were excluded from analyses of health professional skin exams, and those who reported having a skin examination completed by themselves or a non-health professional at the baseline assessment were excluded from analyses of self/partner skin exams. ^‡^ Proportion reporting a skin examination at least once over the 12-month follow-up period.

**Table 4:** Stratum-specific intervention effects for statistically significant moderators.

Outcome	Moderator	Intervention Effect	Intervention Effect P	Moderation P
	*MC1R* Average-Risk			
**Weekend Hours**	**Marital status**			0.019
	Single, separated, divorced, or widowed	−0.15	0.34	
	Married, domestic partnership, or civil union	0.32	0.01	
**Sunburns**	**Family history of non-melanoma skin cancer**			0.049
	No	−0.10	0.07	
	Yes	0.09	0.23	
**Wearing a hat often or always**	**Family history of melanoma**			0.030
	No	1.05 *	0.87	
	Yes	5.23 *	0.01	
**Seeking shade or using umbrella often or always**	**Family history of melanoma**			0.023
	No	0.98 *	0.93	
	Yes	3.67 *	0.01	
	*MC1R* Higher-Risk			
**Sunburns**	**Tendency to burn**			0.032
	Burners	−0.14	0.09	
	Non-burners	0.07	0.16	

* Because component measures of sun protection habits were analyzed as dichotomous outcomes, these effect estimates are expressed as odds ratios.

**Table 5:** Primary prevention outcome measures among children of participants at baseline and post-intervention and intervention effects by the parent’s *MC1R* risk category.

	Standard Arm			Precision Prevention Arm			Intervention Effect *	
Outcomes	Baseline	Post-Intervention ^†^	*p* ^‡^	Baseline	Post-Intervention ^†^	*p* ^‡^	Beta	*p*
*MC1R* Average-Risk								
		*n* = 25			*n* = 21			
**Continuous Outcomes ^§^**								
Weekday hours	1.31	1.29	0.91	1.50	1.23	0.21	−0.06	0.87
Weekend hours	2.23	2.36	0.42	2.29	2.10	0.35	−0.24	0.41
Sunburns	0.31	0.25	0.33	0.48	0.16	0.12	−0.20	0.77
Outdoor intentional tanning	1.09	1.06	0.77	1.13	0.99	0.27	0.09	0.42
**Binary Outcomes ^¶^**								
Wearing a hat often or always **	0.24	0.08	0.26	0.11	0.02	0.19	0.17	0.08
Seeking shade or using umbrella often or always	0.12	0.49	0.15	0.19	0.17	0.87	0.60	0.78
Wearing a shirt with sleeves often or always ^††^	0.85	0.88	--	0.69	1.00	--	--	--
Wearing sunglasses often or always **	0.04	0.02	0.34	0.04	0.04	0.93	1.59	0.81
Wearing sunscreen often or always	0.71	0.70	0.78	0.66	0.44	0.43	0.29	0.24
*MC1R* Higher-Risk								
		*n* = 49			*n* = 46			
**Continuous Outcomes ^§^**								
Weekday hours	1.42	1.34	0.59	1.40	1.47	0.61	0.03	0.38
Weekend hours	2.33	2.28	0.77	2.26	2.24	0.89	0.10	0.76
Sunburns	0.32	0.26	0.33	0.37	0.10	0.01	−0.13	0.03
Outdoor intentional tanning	1.16	1.03	0.02	1.10	1.06	0.20	0.01	0.78
**Binary Outcomes ^¶^**								
Wearing a hat often or always **	0.07	0.07	0.93	0.09	0.05	0.41	0.88	0.87
Seeking shade or using umbrella often or always	0.14	0.07	0.24	0.15	0.18	0.99	2.53	0.10
Wearing a shirt with sleeves often or always	0.91	0.84	0.41	0.84	0.96	0.05	2.45	0.13
Wearing sunglasses often or always ^††^	0.07	0.06	--	0.11	0.12	--	--	--
Wearing sunscreen often or always	0.83	0.69	0.21	0.88	0.86	0.79	2.65	0.06

* Intervention effect compares the post-intervention measure in the intervention arm to that in the standard arm, after adjusting for baseline outcome, season, predictors of missingness, and predictors of the outcome. ^†^ Post-Intervention is the average of outcome measures obtained at the 6- and 12-month assessments. ^‡^ Within arm *p*-values are from tests comparing post-intervention measures to baseline averages from a GEE model containing baseline, 6-, and 12-month outcomes as the dependent variables. **^§^** Baseline and post-intervention values are population predicted marginal means, while the intervention effects are beta-coefficients. ^¶^ Baseline and post-intervention values are population predicted marginal proportions, while the intervention effects are reported as odds ratios (ORs). ** Due to sparse data, estimates for these outcomes are adjusted for baseline season and baseline outcome only. ^††^ Due to zero cells, only raw proportions are reported for these outcomes.

## Data Availability

The data presented in this study are available on request from the corresponding author.

## Supplementary Materials

The following are available online at https://www.mdpi.com/article/10.3390/cancers13133143/s1: Table S1: Minor allele frequency of observed *MC1R* variants and corresponding variant-level risk; Table S2: Additional baseline measures considered as predictors of missingness and outcomes; and Table S3: Baseline predictors of missingness.
